# Drone-Induced Midfacial Blast Injuries: Early Definitive Reconstruction and 5-Year Outcomes from a Single-Center Cohort

**DOI:** 10.3390/jcm15124588

**Published:** 2026-06-12

**Authors:** Anna Poghosyan, Martin Misakyan, Gurgen Mkhitaryan, Davit Minasyan, Irina Malkhasyan, Hayk Petrosyan, Anna Frangulyan, Aren Bablumyan, Armen Minasyan, Armen Muradyan

**Affiliations:** 1 Department of ENT and Maxillo-Facial Surgery, “Heratsi” No 1 Hospital, Yerevan State Medical University, 7 Heratsi Str., Yerevan 0025, Armenia; misakyan86@inbox.ru (M.M.); mfs.ent@ysmu.am (G.M.); deivart@mail.ru (D.M.); 2 Department of Anesthesiology and Intensive Care, “Heratsi” No 1 Hospital, Yerevan State Medical University, 7 Heratsi Str., Yerevan 0025, Armenia; imalkhas@mail.ru (I.M.); dr.haykpetrosyan15031@mail.ru (H.P.); 3 Department of Radiology, “Heratsi” No 1 Hospital, Yerevan State Medical University, 7 Heratsi Str., Yerevan 0025, Armenia; ann.frangulyan@gmail.com; 4 Administrative Department, “Heratsi” No 1 Hospital, Yerevan State Medical University, 7 Heratsi Str., Yerevan 0025, Armenia; heratsi.university.hospital@gmail.com (A.B.); proclinics@ysmu.am (A.M.); rector@ysmu.am (A.M.)

**Keywords:** drone injuries, blast trauma, midfacial fractures, craniomaxillofacial trauma, osteosynthesis, facial reconstruction, military medicine, war surgery, titanium mesh, lipofilling, polytrauma

## Abstract

**Background:** Modern warfare has introduced novel mechanisms of injury, particularly drone-induced blast trauma, resulting in complex craniomaxillofacial injuries. These injuries differ substantially from typical ballistic wounds and require adapted surgical strategies. This study was conducted to evaluate the clinical characteristics, management approaches, and long-term outcomes of midfacial blast injuries. **Methods:** A retrospective analytical study was conducted on 41 patients with drone-induced midfacial blast injuries treated at a tertiary referral center in Armenia following the 2020 Nagorno-Karabakh War. All patients underwent surgical management after initial stabilization and were followed for 5 years. Clinical outcomes, complications, and reconstructive needs were assessed. **Results:** All patients presented with comminuted midfacial fractures, which were frequently associated with polytrauma (87.8%). Burns were observed in 82.9% of cases. Surgical management included radical debridement and early definitive osteosynthesis using titanium fixation systems. No cases of postoperative osteomyelitis, bone sequestration, or implant failure were observed during the 5-year follow-up period. Patients with extensive soft tissue defects, particularly nasal and lip amputations, required multiple reconstructive procedures. Long-term follow-up revealed progressive soft tissue thinning over titanium meshes, especially in the zygomatico-orbital region, necessitating secondary interventions such as lipofilling. **Conclusions:** Drone-induced midfacial blast injuries represent a distinct and severe form of trauma. Early definitive reconstruction following adequate debridement was associated with favorable outcomes. However, soft tissue reconstruction remains challenging and often requires staged procedures. Long-term follow-up is essential to manage delayed complications and optimize aesthetic outcomes.

## 1. Introduction

Current geopolitical tensions have increased the risk of high-intensity military conflicts and state-to-state wars [1].

Recent advancements in military technology have significantly altered the patterns of trauma encountered in modern warfare. In particular, the widespread use of unmanned aerial vehicles (UAVs), especially explosive drones, has introduced a distinct category of high-energy blast injuries [2,3,4]. These injuries differ fundamentally from conventional ballistic trauma due to the combined effects of blast waves, fragmentation, thermal injury, and secondary projectiles [1,3,5].

The management of cranio-maxillofacial trauma (CMFT) in war-torn nations presents a formidable challenge precipitated by limited resources, damaged infrastructure, and a dearth of skilled surgical professionals [6]. Injuries to the craniofacial region are of particular concern due to the high density of critical anatomical structures, including the airway, major vascular pathways, sensory organs, and functionally important soft tissues [1].

In this context, midfacial trauma presents unique diagnostic and therapeutic challenges. The combination of comminuted fractures, extensive soft tissue loss, orbital and cranial injury, contamination, and compromised vascularity—often exacerbated by delayed evacuation or suboptimal initial care—complicates both acute management and reconstructive planning [7]. Despite the increasing prevalence of such injuries, the current literature remains limited in systematically characterizing their clinical features and defining optimal management strategies, particularly for head and neck involvement. Most existing studies focus on conventional ballistic trauma or generalized blast injuries, with insufficient emphasis on the specific injury patterns and reconstructive challenges associated with modern drone-related mechanisms.

To our knowledge, this is one of the first studies specifically addressing the long-term outcomes of drone-induced midfacial blast injuries.

The aim of this study was to analyze the clinical features, surgical management, and long-term outcomes of midfacial blast injuries in a cohort of patients treated during a recent military conflict.

## 2. Materials and Methods

### 2.1. Study Design and Setting

This retrospective analytical study was conducted at the Department of Otorhinolaryngology and Maxillofacial Surgery at “Heratsi” hospital, Yerevan, Armenia. Military patients wounded in the 2020 Nagorno-Karabakh War who were treated between September and November 2020 were included in the study. This research was conducted in accordance with relevant ethical standards, and the study protocol was approved by the Yerevan State Medical University Ethics Committee: IRB Expert Conclusion No. YSMU № 9/17-18; Date: 5 March 2026.

#### 2.1.1. Ethical Considerations

Ethical considerations were maintained throughout the study, and the patients’ names and medical information were kept completely confidential. The medical histories of the patients were used solely for the purposes of this study.

#### 2.1.2. Patient Selection

The inclusion criteria were drone-induced blast injuries involving the midface and complete medical records and 5-year follow-up data. The exclusion criteria were injuries due to non-blast mechanisms (e.g., gunshot wounds, blunt trauma), isolated soft tissue injuries without midfacial skeletal involvement, and incomplete data or loss to follow-up.

#### 2.1.3. Study Population

A total of 43 patients with drone-induced midfacial blast injuries were initially screened for eligibility. Two patients were excluded due to incomplete long-term follow-up data. Therefore, 41 patients were included in the final analysis. The STROBE-compliant patient selection process is illustrated in the Supplementary Materials. All included patients were male and aged 18–46 years (mean age: 25.3 years).

#### 2.1.4. Injury Characteristics

Due to the high-energy nature of blast trauma, the majority of injuries demonstrated severe comminuted multifragmentary fracture patterns involving multiple midfacial compartments simultaneously. Because these injuries frequently crossed conventional anatomical boundaries, fracture patterns were classified according to a topographic midfacial classification system, including central midface fractures (Le Fort I–II, sagittal maxillary fractures, and nasoethmoidal fractures), centrolateral fractures (Le Fort III and zygomatico-orbital fractures), lateral fractures (zygomaticomaxillary complex and zygomatic arch fractures), and combined multifragmentary midfacial fractures [8]. Naso-ethmo-orbital (NOE), zygomaticomaxillary complex (ZMC), and zygomatico-orbital complex (ZOC) injuries were the predominant fracture patterns within the cohort.

#### 2.1.5. Examinations

The patients underwent 5 years of follow-up, including clinical and radiological evaluation. Postoperative skin and soft tissue thinning under titanium meshes was evaluated clinically according to the severity of flap atrophy and the risk of implant exposure. Grading was generally based on objective tissue thickness and exposure status. For precise diagnostic grading and head structure assessment, high-resolution CT scans were used to evaluate postoperative hard and soft tissues.

Comminuted midfacial fractures were observed in 41 patients. Associated mandibular fractures were present in 11 (26.8%; *n* = 41) cases. (Table 1) Combined skull-base injuries were present in five (12.2%; *n* = 41) patients. Four patients had severe orbital injuries requiring evisceration. Additional injuries included nasal amputation (*n* = 2), lip defects (*n* = 3), and facial burns of varying severity (*n* = 34; 82.9%); 36 patients had combined and multiple (polytrauma) injuries. Pure tone audiometry (PTA) over the standard 0.250–8 kHz range and at 12 kHz, as well as distortion product otoacoustic emission (DPOAE) and auditory brainstem response (ABR) testing, were performed for the assessment of blast-related acoustic neuritis [9].

#### 2.1.6. Triage and Surgical Management

Drone-related explosive injuries during active military conflict generated mass casualty conditions requiring rapid triage and coordinated multidisciplinary management. Initial priorities included airway protection, hemorrhage control, circulatory stabilization, and damage-control procedures. During the forward evacuation stages, the wounded personnel received emergency surgical wound revision and resuscitative treatment in field hospitals or nearby military medical facilities before they were transferred to tertiary referral centers.

**Figure 1:**
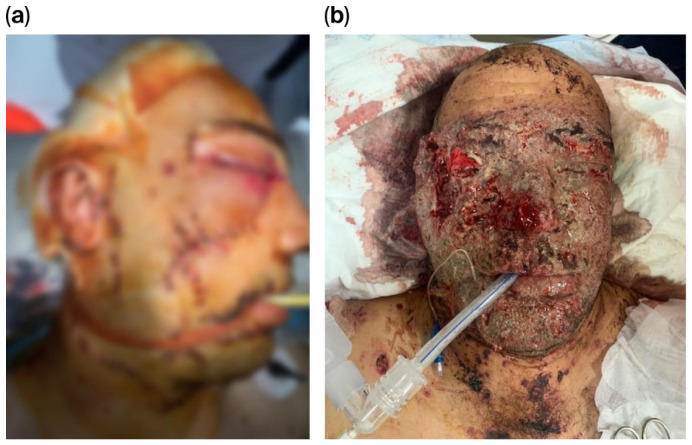
Initial clinical presentation of a patient with severe comminuted midfacial blast injury upon admission to the intensive care unit: (**a**) Drone-blast-injured patient admitted with primary surgical wound debridement; initial hemodynamic and respiratory stabilization. Multiple perforating wounds are observed. (**b**) Drone-blast-injured patient with severe III^0^–IV^0^ burns admitted with primary surgical wound debridement; initial hemodynamic and respiratory stabilization.

Following tactical and strategic medical evacuation, the patients were transferred to specialized hospitals located outside the active combat zone. Our institution was one of the tertiary referral centers for definitive craniofacial reconstruction. Upon admission, all patients initially underwent intensive care monitoring and comprehensive multidisciplinary assessment (Figure 1).

Documentation accompanying the evacuated patients included information on prior surgical interventions, vaccinations, imaging studies, medications, and procedures performed during earlier evacuation stages. Definitive surgical planning was subsequently individualized according to hemodynamic status, associated injuries, soft tissue condition, and radiological findings.

Of the 41 patients with comminuted midfacial blast injuries, 17 (41.5%; *n* = 41) were admitted intubated under mechanical ventilation. All patients underwent surgical treatment following initial stabilization. The timing of surgical management was influenced by injury severity, surgical urgency, and the presence of concomitant injuries (including thoracic trauma and traumatic brain injury), which required continuous monitoring and hemodynamic stabilization. It is important to note that, as the military operations occurred during the COVID-19 pandemic, all hospitalized wounded patients were routinely screened for the virus. Patients with positive test results were isolated in dedicated intensive care units. In COVID-19-positive cases, chest computed tomography (CT) was performed systematically, and in the presence of pneumonia, appropriate treatment was initiated. Whenever feasible, surgical intervention was postponed until clinical stabilization. Among patients with midface injuries, only one patient tested positive for COVID-19 in the preoperative period and remained asymptomatic. Following a negative test result one week later, the patient subsequently underwent surgical treatment.

The interval between hospital admission and definitive osteosynthesis in this cohort ranged from 0 to 12 days, depending on hemodynamic stability, associated injuries, intensive care requirements, and COVID-19-related organizational factors. The mean time to definitive reconstruction was 1.66 ± 2.32 days, with a median interval of 1 day. Most patients underwent definitive reconstruction within the first 72 h after admission, following adequate stabilization and wound assessment.

**Figure 2:**
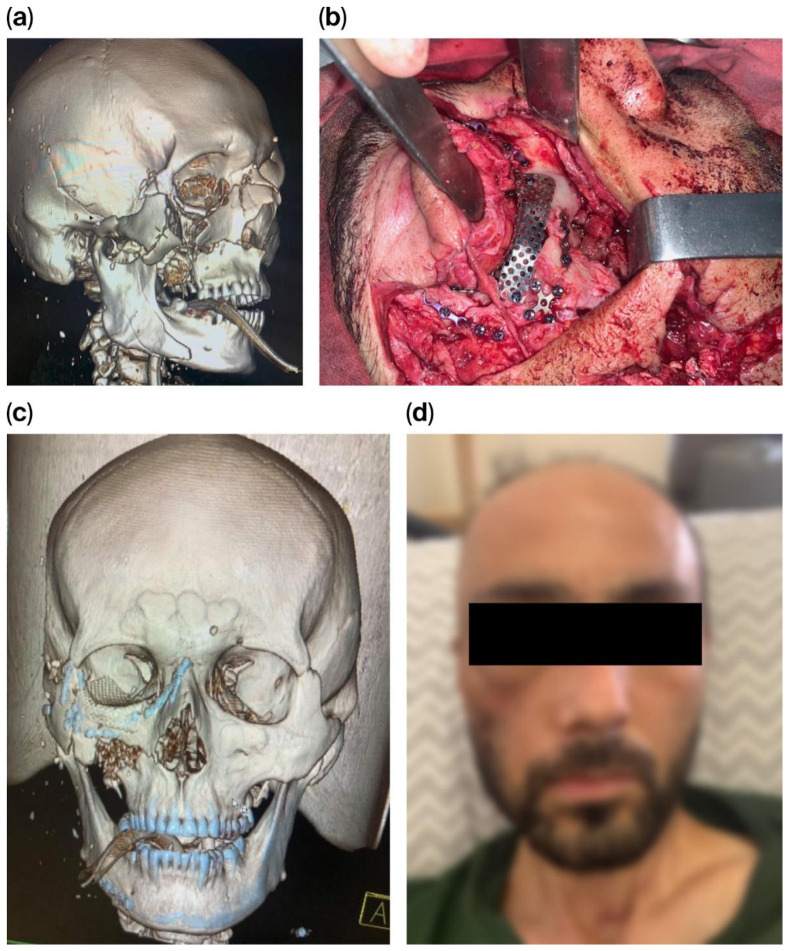
Drone-blast midfacial injury: (**a**) 3D CT scan demonstrates a right-site severely comminuted midface fracture; (**b**) early definitive osteosynthesis and blow-out reconstruction using titanium mesh and plates; (**c**) postoperative 3D CT scan; (**d**) long-term (5-year follow-up) postoperative result demonstrating soft tissue thinning over titanium mesh and plates in the right zygomatic region.

**Figure 3:**
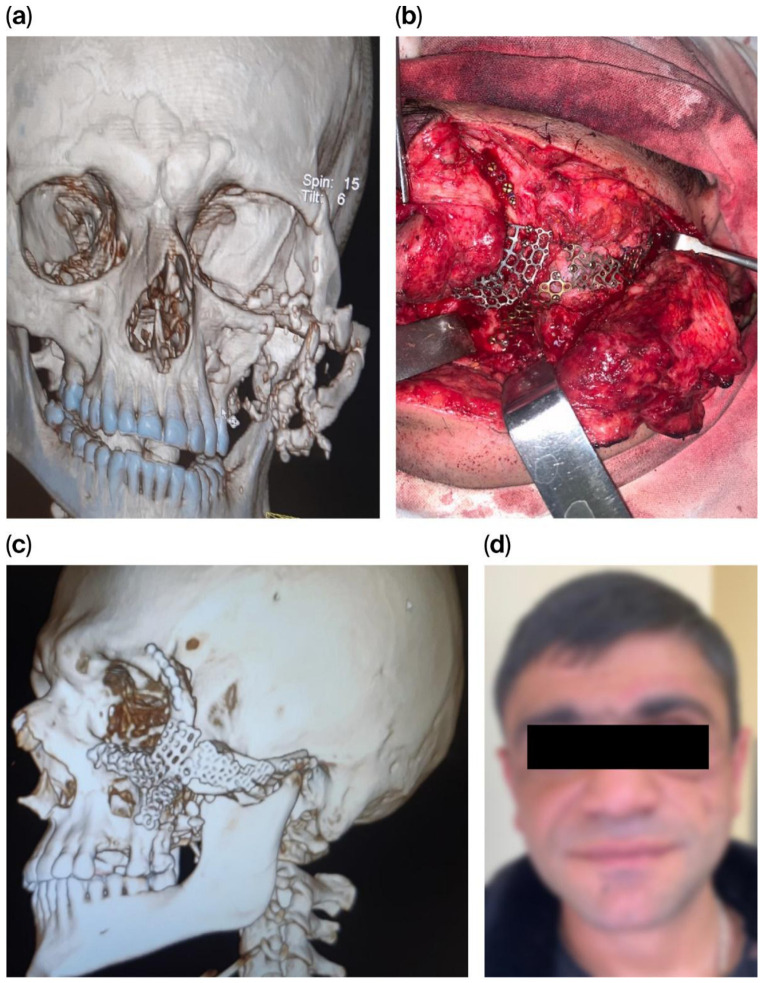
Drone-blast midfacial injury: (**a**) 3D CT scan demonstrates a left-site severely comminuted midface fracture; (**b**) early definitive reconstruction osteosynthesis using titanium mesh and plates; (**c**) postoperative 3D CT scan; (**d**) long-term (5-year follow-up) postoperative result demonstrating soft tissue thinning over titanium mesh and plates in the left zygomatic region.

Management included radical debridement of necrotic tissues, final rigid osteosynthesis, and reconstruction of bone defects using titanium plates and meshes where required. Multiple soft tissue injuries were characteristic of drone blast injuries. The reconstructive strategy was individualized based on defect extent and tissue condition (Figure 2 and Figure 3).

### 2.2. Follow-Up and Outcomes

The patients were followed for 5 years postoperatively. Outcome measures included structural stability, wound healing, complications, functional recovery, and aesthetic results. Secondary procedures were recorded.

### 2.3. Statistical Analysis

Descriptive statistics were used to summarize the clinical data. Continuous variables are presented as mean values, and categorical variables as frequencies and percentages. In addition to descriptive statistics, subgroup analyses were performed to explore associations between injury characteristics and clinical outcomes. Categorical variables were compared using the chi-square test and Fisher’s exact test. A *p*-value < 0.05 was considered statistically significant. Statistical analyses were performed using SPSS, version 16.0 (SPSS Inc., Chicago, IL, USA).

## 3. Results

### Postoperative Outcomes and Complications

**Figure 4:**
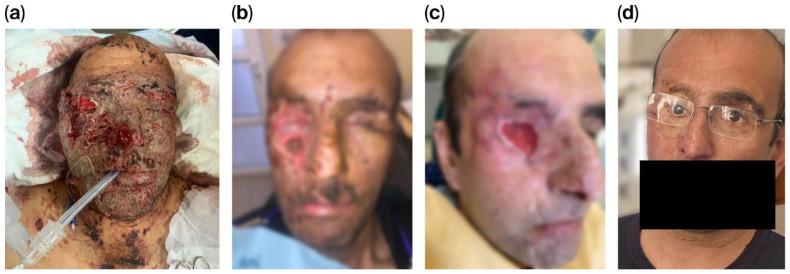
Burn-associated soft tissue injury with delayed healing requiring necrectomy and local flap reconstruction: (**a**) drone-blast-injured patient with severe III^0^–IV^0^ burns and comminuted right-site midface fracture; (**b**) one month follow-up after wound debridement, left eye evisceration, right eye enucleation, and zygomatico-orbital osteosynthesis, healing under iodoform gauze; (**c**) 3-month follow-up after multiple local soft tissue flap reconstruction; (**d**) patient with prosthetic rehabilitation after 5 years of follow-up.

Burn-associated injuries were associated with delayed healing and required prolonged wound care, including healing under iodoform gauze dressings, surgical necrectomy, and local flap reconstruction (Figure 4).

Subgroup analysis was performed to evaluate the impact of burn injuries and soft tissue defects on clinical outcomes (Table 2).

Patients with burn-associated injuries required secondary reconstructive procedures significantly more frequently than patients without burns (28/34 [82.4%] vs. 1/7 [14.3%]; Fisher’s exact test, *p* = 0.0012; χ^2^ = 12.99, df = 1, *p* = 0.0003; OR = 28.0, 95% CI: 2.83–277.42).

No statistically significant differences were observed in the rates of postoperative infection or implant-related complications between subgroups.

Four patients (9.7%; *n* = 41) underwent evisceration due to severe eyeball injury; in one patient, both eyes were eviscerated. No cases of postoperative osteomyelitis or bone sequestration were observed during the 5-year follow-up period. All fracture reconstructions demonstrated stable outcomes without implant-related infection or failure. The patients with nasal amputation and large lip defects required more surgical interventions for functional and esthetic reconstruction (Table 3).

The long-term outcomes (5 years) demonstrated progressive thinning of soft tissues over titanium meshes, resulting in esthetic imbalance, especially in the zygomatico-orbital zone (Figure 2d and Figure 3d).

In our cohort, no titanium plate or mesh exposure (extrusion) was observed. Overlying skin thinning and “skeletonization” of the hardware were observed in all patients with zygomatico-orbital fractures.

**Figure 5:**
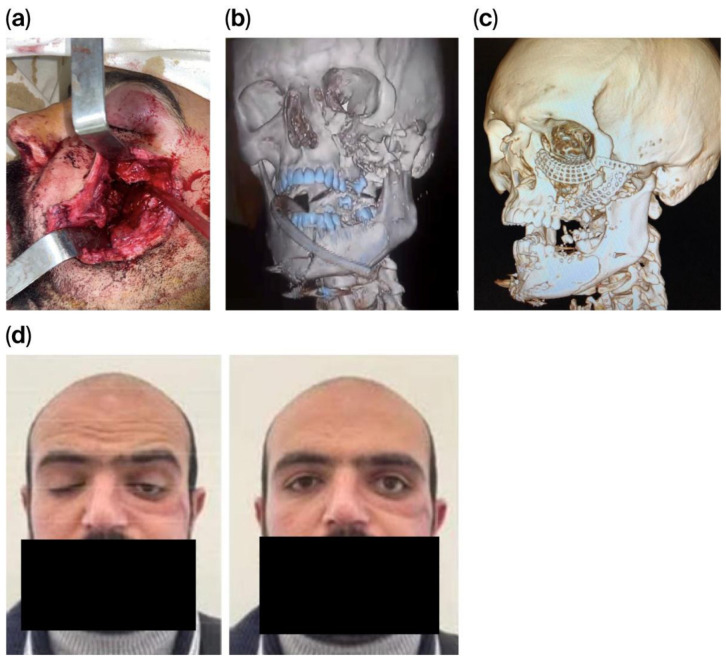
Drone-blast open midfacial and mandible injury: (**a**) 3D CT scan demonstrates left-site severely comminuted multifragmental midface (zygomatico-orbital, NOE, and zygomatico-maxillary complex) and mandible fractures; (**b**) early definitive wound access reconstruction osteosynthesis using titanium mesh and plates; (**c**) postoperative 3D CT scan; (**d**) long-term (5-year follow-up) postoperative result demonstrating soft tissue thinning over titanium mesh and plates in the left zygomatic region; lower left eyelid deficit malocclusion because of second upper left molar disposition.

Post-traumatic malocclusion was observed in one patient due to secondary displacement of the upper second molar after reconstruction; however, the patient refused further corrective treatment. Infraorbital nerve persistent deficits were observed in six (14.6%; *n* = 41) patients with combined multifragmental zygomatico-orbital and zygomatico-maxillary fractures over the 5-year follow-up period. Restricted ocular motility and lower eyelid deficit were observed in two patients with open zygomatico-orbital fractures and facial nerve branch injuries (Figure 5a–d).

Secondary procedures, including lipofilling, were required to restore soft tissue volume and improve facial symmetry in certain cases.

Due to the specific cultural and psychological characteristics of this patient population, none of the patients complained about impaired masticatory efficiency. Nevertheless, objective examination revealed secondary edentulism in 28 out of 41 patients. It should be emphasized that none of these patients had consulted a dentist for prosthetic rehabilitation of the dental arch.

Only 16 patients underwent oral treatment procedures, including treatment of dental caries or pulpitis and professional cleaning. The remaining patients demonstrated poor oral hygiene. Post-traumatic malocclusion was observed in one patient due to a premature occlusal contact of the upper second molar caused by post-reconstructive dystopia; however, the patient refused selective grinding or extraction of the tooth.

One patient is still undergoing rehabilitation because of the severity of the war injury and, at the time of the follow-up examination, remained with a tracheostomy, gastrostomy, spinal cord injury, and post-traumatic epileptic seizures requiring anticonvulsant therapy.

**Figure 6:**
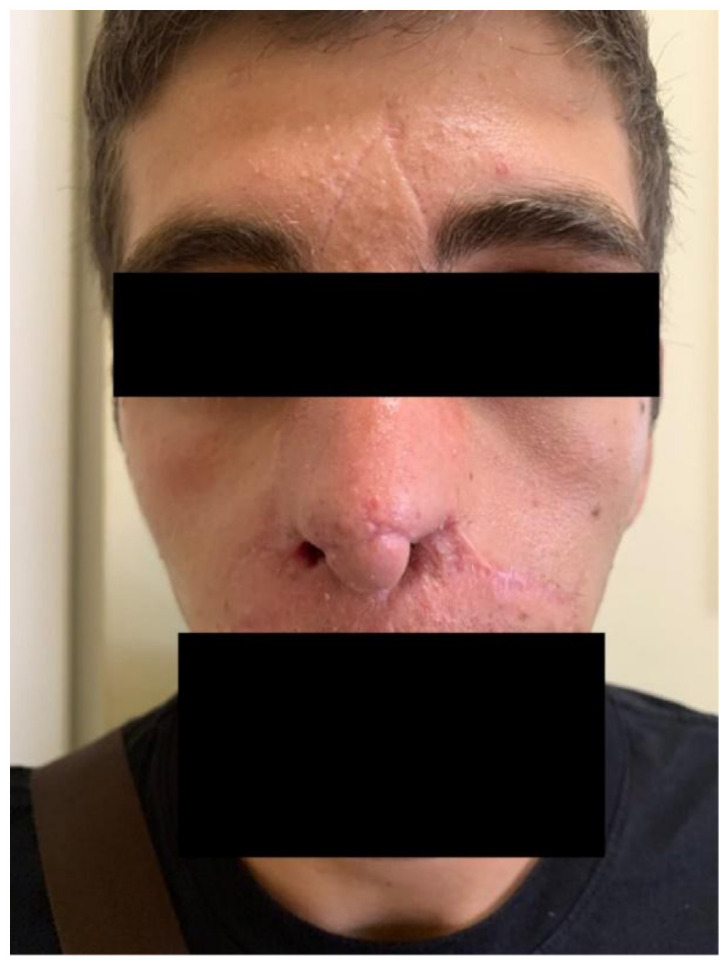
Nasal tip scarring and nasal passage narrowing in patient after nasal tip amputation and subsequent 3-step flap surgery.

On the 5-year follow-up CT scans, 36 out of 41 patients demonstrated nasal septum deviation and hypertrophic rhinitis. However, none complained of impaired nasal breathing or agreed to undergo septoplasty. Only one patient, who had sustained nasal tip amputation and underwent a three-stage reconstruction, reported nasal breathing difficulties. Objective examination revealed severe narrowing of the nasal passages due to scarring and flap reconstruction procedures (Figure 6). Representative staged reconstructive timelines of the most severe injuries are summarized in Supplementary Table S1.

Many patients demonstrated limited interest in secondary corrective procedures despite objective functional or esthetic deficits, possibly reflecting psychological adaptation after severe wartime trauma. None of the patients from this cohort received psychological counseling after hospital discharge.

**Figure 7:**
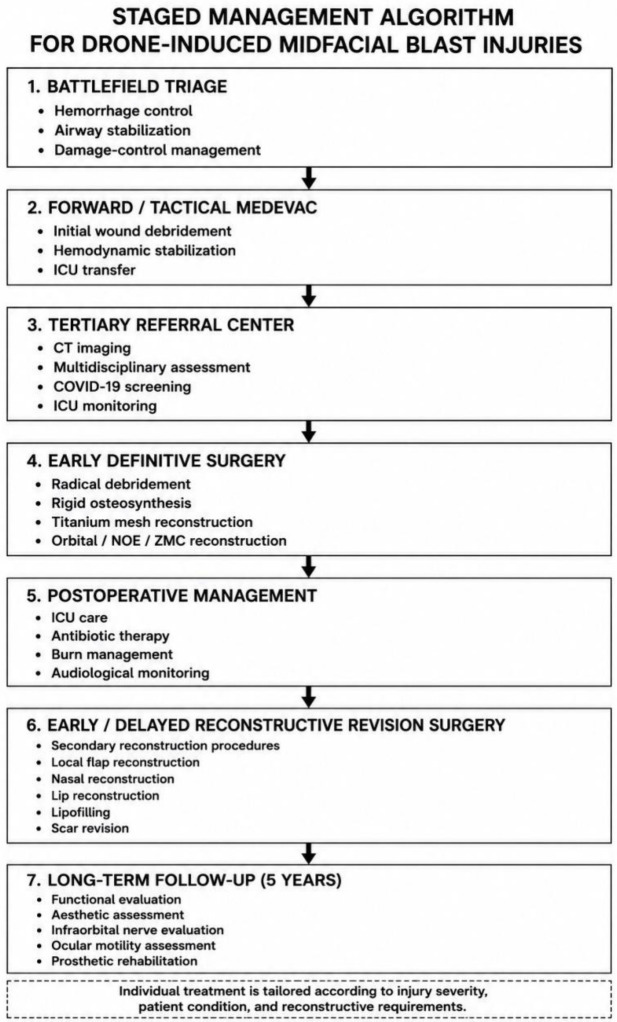
Staged treatment algorithm for drone-induced midfacial blast injuries.

Clinical and audiometric findings consistent with blast-related acoustic neuritis were identified in all patients. The most common manifestations included tinnitus, high-frequency sensorineural hearing impairment, and subjective hearing reduction. Conservative ENT management included systemic corticosteroid therapy, neuroprotective treatment, vasoactive medications, and long-term audiological follow-up. A staged management algorithm proposed for drone-induced midfacial blast injuries is provided in Figure 7.

## 4. Discussion

This study demonstrates that drone-induced midfacial blast injuries are characterized by severe comminuted multifragmentary fractures frequently associated with burns, orbital trauma, and polytrauma. Early definitive reconstruction following adequate debridement resulted in stable long-term osseous outcomes without implant-related infection or osteomyelitis. However, delayed soft tissue atrophy and secondary esthetic deformities, particularly in the zygomatico-orbital region, remained important long-term reconstructive challenges.

On 27 September 2020, Azerbaijan initiated a large-scale war against Nagorno-Karabakh. As a result of these unrelenting attacks, large numbers of people have been wounded and require medical care, which has subjected the Armenian and Nagorno-Karabakh healthcare systems to unprecedented pressure. This strain has forced many of the existing COVID-19 centers to shift their scope, and most non-emergency medical care has either been delayed or cancelled [10].

The increasing use of unmanned aerial systems in modern warfare has substantially altered the pattern of combat-related injuries. Drone-deployed explosive devices frequently cause combined blast, fragmentation, and thermal injuries involving exposed craniofacial structures. Unlike conventional ballistic trauma, these mechanisms often produce multifocal tissue destruction, multiple perforating wounds, extensive contamination, and irregular comminuted fractures. In addition to direct kinetic damage, blast-wave overpressure and thermal exposure contribute to complex soft tissue injury patterns and delayed reconstructive challenges. These combined mechanisms likely explain the high frequency of burns, orbital trauma, and associated polytrauma observed in our cohort [11,12,13,14].

In contrast to isolated gunshot wounds, drone-related injuries are typically caused by a combination of blast-wave barotrauma, high-velocity fragmentation, thermal injury, and secondary projectiles. Furthermore, drone-deployed explosive devices frequently detonate in close proximity to exposed craniofacial structures during trench warfare, producing multiple perforating wounds and combined soft tissue–skeletal destruction. Additionally, the use of miniaturized explosive ordnance and improvised metallic fragments may contribute to diffuse tissue cavitation, extensive contamination, and irregular multifragmentary fracture patterns. These mechanisms likely explain the high prevalence of combined burns, polytrauma, orbital injuries, and complex reconstructive requirements observed in our cohort.

Gumeniuk KV et al. (2021) highlighted the key distinctive features of drone-blast induced injuries, which include multiple concomitant and combined types of damage (mechanical, thermal, and chemical) [15].

Facial combat injuries are high-energy, war-related maxillofacial traumas resulting from blast, ballistic, or fragmentation mechanisms, typically characterized by combined osseous and soft tissue destruction, contamination, and frequent association with multisystem injuries. These are injuries that cause cosmetic, functional, and psychological damage [15,16]. As reported by Wordsworth et al. (2017), in 75% of blast injury survivors, explosive devices were the most common sources of injury, and the midface was the most commonly affected facial region [17]. In blast injuries, facial fracture is a significant factor in an increased total injury severity score [3,17]. Many authors have reported that the incidence of midfacial injuries is increasing. Keller et al. (2015) found that the overall incidence of head and neck trauma in modern combat has increased from 6% to 21% in early conflicts (e.g., World War II and the Korean War) to 21% to 43% in more recent conflicts (e.g., the Somali civil war, Operation Iraqi Freedom, and Operation Enduring Freedom) [18]. In contrast, during the Syrian Civil War, 66.3% of trauma casualties were caused by gunshot wounds and 31.3% by blast injuries, with facial injuries being the second most common type after extremity injuries [19].

Prysiazhniuk O et al. (2025) [3] reported that the primary cause of war-related maxillofacial injuries in the Russo-Ukraine war is high-energy blast trauma resulting from artillery strikes, mines, drones, rocket attacks, and bombings. War-related military trauma involved soft tissue damage in 97.1% of cases. This is in accordance with our data, as only two patients with gunshot injury were hospitalized in our department, and they were not included in this study.

Efficient and well-structured medical evacuation of the wounded is a key component in ensuring effective medical care during mass casualty situations and military operations [7,20]. The military medical evacuation (MME) system is an intricate network of interconnected teams providing a range of medical treatments from the point of injury (POI) to healthcare facilities in the patient’s home country or another secure place outside of the joint operations area [20,21]. The MME system/enterprise is complex. Safe and rapid evacuation of those wounded in combat to facilities capable of providing the appropriate level of care is the key concern of any emergency medical evacuation (MEDEVAC) team [20]. There are three main categories of MEDEVAC: forward, tactical, and strategic. In forward MEDEVAC, the patient is transported as soon as possible from the site of injury to the most appropriate care facility, not necessarily the nearest care facility. Accordingly, triage of patients plays a key role in this process. During forward MEDEVAC, it is of paramount importance that the patient is provided appropriate care according to the 10-1-2 Timeline, a set of predetermined clinical time frames (i.e., controlling bleeding within 10 min, advanced resuscitative care within 1 h, and damage control surgery within 2 h). Tactical MEDEVAC concerns the transport of patients between different MTFs; generally, care levels increase from lower to higher. This type of evacuation occurs within a Joint Operational Area. Strategic MEDEVAC is the movement of ill or wounded personnel from the Joint Operational Area to a facility in their own country or to another safe location outside the theater [20,22].

Armenia gained experience in medical evacuation during previous military conflicts in Nagorno-Karabakh between 1991 and 1994 and in 2016. From the beginning of the 2020 Nagorno-Karabakh War, many highly qualified physicians from the Armenian diaspora, including maxillofacial surgeons, were voluntarily deployed to the Nagorno-Karabakh and border regions of Armenia, in close proximity to active combat zones. Their role was to provide urgent and specialized care and to organize the medical evacuation of the wounded in accordance with the aforementioned MEDEVAC system. As a result of this coordinated approach, patients arriving at definitive care facilities, including our hospital, presented with appropriately managed wounds and stable hemodynamic and respiratory status.

The management of wartime injuries during the COVID-19 pandemic represented an additional organizational and surgical challenge. COVID-19 screening was performed only during tactical and strategic medical evacuation when the patient was hospitalized in a transitional or final specialized hospital. In our hospital, separate intensive care units were established for COVID-19-negative patients, COVID-19-positive patients, and newly admitted patients with unknown infection status. Patients with confirmed COVID-19 infection were generally managed conservatively or by minimally invasive intervention whenever feasible, with surgical intervention under general anesthesia delayed until clinical stabilization and negative testing. In this cohort, only one patient experienced delayed definitive reconstruction because of a positive COVID-19 test result. He had an open wound, which was sutured during forward medical evacuation. After seven days of delaying surgery, the wound healed, requiring a new incision in the same wound. No adverse long-term reconstructive or infectious outcomes associated with this delay were identified. 

High-energy ballistic and avulsion injuries to the face represent some of the most complex challenges in modern reconstructive surgery [5]. Maxillofacial injuries comprise a significant proportion of war-related injuries, and their characteristics have evolved over decades as new types of weapons and battlefield strategies have been introduced [2,3,14,18,23,24].

Prysiazhniuk O et al. reported that the predominance of high-velocity blast injuries in their cohort was associated with concomitant maxillofacial trauma to other organs and systems in 82% of patients [3]. The most frequently associated injuries were to the extremities (49.9%) and the brain (32%), both of which significantly impacted prognosis and posttraumatic outcomes. Ophthalmic trauma, which is frequently associated with midfacial fractures and defects, is another major concern; 50.1% of maxillofacial trauma patients in this study suffered eye injuries, with 9.2% experiencing eye loss [3]. In this study, all patients with maxillofacial trauma had concomitant injury to other organs and systems. Extremity injuries were the most frequently associated injuries and were found in 78% of cases. Eyeball injuries with associated evisceration were reported in 9.8% of patients, which is in accordance with data from Prysiazhniuk O et al. (2025) [3]. In the Syrian War, approximately 63% of maxillofacial injuries were accompanied by extremity or other organ injuries, reflecting the widespread impact of shrapnel, missiles, and bullets [25]. Burns were observed in 82.9% of cases in this study, which aligns with most blast injury studies [3,5,6,14,18]. The subgroup analysis further supports the clinical observation that burn-associated injuries and extensive soft tissue defects are key determinants of delayed healing and the need for secondary reconstruction. These findings highlight the importance of individualized treatment strategies and reinforce the role of early identification of high-risk patients. The auditory system is particularly vulnerable to blast-wave overpressure, resulting in cochlear hair cell damage, neural pathway injury, and central auditory dysfunction. In the present cohort, all patients demonstrated clinical and audiometric findings consistent with acoustic neuritis following drone-induced blast exposure. These findings are consistent with previously published military studies reporting high rates of hearing impairment among soldiers exposed to explosive and blast-related trauma. MacGregor et al. reported auditory dysfunction in 92.2% of military personnel exposed to blast injuries during conflicts in Iraq and Afghanistan [26]. Our findings further emphasize the importance of multidisciplinary management and long-term otorhinolaryngological follow-up in patients with severe craniomaxillofacial war trauma.

Most current evidence regarding the management of high-energy craniofacial blast trauma remains limited to retrospective series and military experience reports. Consequently, optimal timing of reconstruction, the extent of debridement, and sequencing of secondary reconstructive procedures remain incompletely standardized. In our cohort, early definitive osteosynthesis following adequate stabilization and wound revision resulted in favorable long-term skeletal outcomes without increased rates of infection or hardware failure [5]. Early definitive reconstruction remains one of the principal controversies in the management of high-energy craniofacial war injuries. In this cohort, definitive osteosynthesis was performed within a relatively early treatment window (1.66 ± 2.32 days), following adequate stabilization and debridement. These findings support the feasibility and relative safety of early definitive reconstruction in select patients with severe drone-induced midfacial blast injuries.

Only patients with nasal and lip amputations and one patient with severe combined facial burns (III^0^–IV^0^) underwent several-step operations for soft tissue reconstruction. Pepper T. et al. (2026) encouraged surgeons to move away from cautious, traditional methods of management with delayed reconstruction and instead favor treatment with earlier internal fixation, emphasizing the need for close attention to wound bed preparation to increase the chance of success [5].

All fracture reconstructions described in this study demonstrated stable outcomes without implant-related infection or failure. No cases of postoperative osteomyelitis or bone sequestration were observed during the 5 years of follow-up. This is in accordance with Pepper et al.’s consensus recommendation, in which it was reported that early definitive bony reconstruction within 10–14 days leverages the window during which the soft-tissue envelope remains extensible and regional vascularity favors graft/flap perfusion. Delays beyond this period increase scar contracture, distort facial buttresses and occlusion, and complicate both surgical access and esthetic outcomes.

In this study, the long-term outcomes (5 years) demonstrated progressive thinning of soft tissues over titanium meshes, resulting in esthetic imbalance, especially in the zygomatico-orbital zones. This may occur due to chronic irritation, pressure necrosis, foreign body reaction, and reduced vascularity; key risk factors are thin overlying soft tissue, infection, and comorbidities [27,28]. Autologous fat grafting (lipofilling) is increasingly recognized as an effective adjunctive technique for the correction of post-traumatic facial asymmetry, soft tissue deficiency, and contour irregularities after craniomaxillofacial reconstruction [29]. In our cohort, lipofilling was successfully used to correct delayed zygomatic soft tissue atrophy in one patient; most patients declined additional esthetic revision procedures despite objective deformities. Further prospective and multicenter studies are required to validate these findings and to develop standardized treatment protocols.

## 5. Study Limitations

This study has several limitations. First, the retrospective single-center design limits the generalizability of the findings. Second, the cohort consisted exclusively of male military patients, which may reduce the findings’ applicability to civilian or female populations. Third, the relatively small sample size, particularly within subgroup analyses, limits statistical power. In addition, validated patient-reported outcome instruments and standardized injury severity scoring systems such as the Facial Injury Severity Scale (FISS) or Injury Severity Score (ISS) were not consistently available because of wartime conditions and the retrospective nature of data collection. The absence of a comparison group also limited the direct evaluation of alternative reconstructive strategies. Validated patient-reported outcome instruments, such as FACE-Q, and standardized facial trauma scoring systems, including FISS, were not consistently applied because of the retrospective nature of this study and wartime data collection conditions. Finally, long-term psychological outcomes were not formally assessed despite the significant psychosocial burden associated with severe facial war injuries.

## 6. Conclusions

Modern warfare imposes a profound burden on society, particularly for young individuals who face the long-term functional and psychosocial consequences of trauma. Drone-induced blast injuries of the midface have distinct characteristics compared to conventional gunshot injuries and can often be managed with early definitive reconstruction, allowing for restoration of anatomical integrity. However, soft tissue defects—especially of the nasal and perioral regions—remain challenging and frequently require staged reconstruction.

From a clinical perspective, early surgical management combined with individualized reconstructive planning is essential. Long-term follow-up is critical for addressing soft tissue deficiencies and optimizing aesthetic outcomes. Future research should focus on developing standardized treatment protocols and improving reconstructive strategies to enhance long-term functional and psychosocial recovery.

## Figures and Tables

**Table 1:** Distribution of midface fractures—combined and comminuted injuries.

Midface Fractures: Combined and Comminuted Injuries (*n* = 41)	*N* (%)
Skull-base fracture	5 (12.2%)
Mandible fracture	11 (26.8%)
Eyeball injury with evisceration	4 (9.8%)
Injury to extremities	32 (78%)
Thoracic or other trunk injuries	4 (9.8%)
Burns	34 (82.9%)

**Table 2:** Comparison of clinical outcomes based on injury characteristics.

Clinical Course	Burn Group (*n* = 34)	No Burn (*n* = 7)	*p*-Value
Healing time (range, median)	12–60 days (17.5 ± 1.2)	7–10 days (8.7 ± 0.4)	0.0003
Secondary procedures or surgeries	28	1	0.0012
Infection	None	None	NS

**Table 3:** Secondary soft tissue surgical procedures performed for functional and esthetic recovery in patients with midface mine-blast injuries.

Diagnosis	Necrectomy	Local Flap Reconstruction	Skin Graft	Lipofilling
1. Right-site multifragmented ZO fracture; whole-face burns of III^0^–IV^0^; polytrauma	4 times	8 times	0	0
2. Combined left-site open NOE multifragmental fracture; nasal tip amputation, I–II^0^ burns; polytrauma	1 time+ wound dressing	2 times	0	0
3. Combined NOE multifragmental fracture; nasal tip amputation; I–II^0^ burns; polytrauma	2 times+ wound dressing	4 times	0	0
4. Left-site combined ZO; ZM multifragmental fracture; polytrauma	0	0	0	Left-site zygoma region lipofilling
5. Combined left-site NOE fracture; upper lip full-thickness wound with 2–2.5 cm defect; crown fracture in upper frontal teeth; I^0^ burns	0	2	0	0
6. Combined right-site multifragmental ZM fracture; cheek and upper lip full-thickness wound with 1.5–2 cm defect; I^0^ burns	0	2	0	0
7. Combined right-site multifragmental ZM fracture associated with mandible body and open fracture of parasymphisis; lower lip full-thickness wound with 3–3.5 cm defect; I^0^ burns	0	3	0	0
8. Other 25 cases of combined midfacial fractures associated with I–II^0^ burns	0 necrectomies Only eschar debridement; local conservative treatment with antibacterial and keratoplastic ointment dressings	0	0	0

## Data Availability

Data are available from the corresponding author upon reasonable request.

## Supplementary Materials

The following supporting information can be downloaded at: https://www.mdpi.com/article/10.3390/jcm15124588/s1, Figure S1: The STROBE-compliant patient selection; Table S1: Representative reconstructive timelines in patients with severe drone-induced midfacial blast injuries; Table S2: STROBE Checklist for Observational Cohort Studies.

## Abbreviations

The following abbreviations are used in this manuscript:

CT	Computed tomography
MME	Military medical evacuation
MEDEVAC	Medical evacuation
