# Supplementary material for: Drone-Induced Midfacial Blast Injuries: Early Definitive Reconstruction and 5-Year Outcomes from a Single-Center Cohort
Source: J Clin Med. 2026 Jun 12;15(12):4588. doi: 10.3390/jcm15124588 (PMC13301518; doi:10.3390/jcm15124588)
Supplement: Supplementary file 1 [file jcm-15-04588-s001.zip › Supplementary Table S1.pdf]

Supplementary Table S1.

Representative reconstructive timelines in patients with severe drone-induced midfacial blast injuries

Patient / Injury Pattern	Initial Definitive Surgery	Early Postoperative Period	Secondary Reconstruction	Long-Term Follow-Up
Severe zygomatico-orbital multifragmentary fracture with III–IV degree facial burns	Radical debridement + titanium mesh osteosynthesis	ICU treatment, prolonged wound healing<br>under iodoform gauze	Multiple necrectomies and local flap reconstructions	Persistent soft tissue atrophy; prosthetic rehabilitation
NOE fracture with nasal tip amputation and burns	Early osteosynthesis + soft tissue repair	Wound dressings and scar management	Multistage nasal flap reconstruction	Nasal airway narrowing and scar-related deformity
Combined zygomaticomaxillary fracture with upper lip full-thickness defect	Definitive osteosynthesis + primary soft tissue repair	Monitoring of flap viability	Local flap reconstruction	Stable oral competence and acceptable aesthetic outcome
Severe zygomatico-orbital fracture with delayed soft tissue atrophy	Titanium mesh orbital reconstruction	Uneventful osseous healing	Lipofilling procedure	Improved facial contour symmetry
Combined NOE and orbital fracture with ocular trauma	Definitive osteosynthesis + orbital reconstruction	Ophthalmologic monitoring	Prosthetic rehabilitation after evisceration	Persistent ocular functional deficit
