# Supplementary material for: Drone-Induced Midfacial Blast Injuries: Early Definitive Reconstruction and 5-Year Outcomes from a Single-Center Cohort
Source: J Clin Med. 2026 Jun 12;15(12):4588. doi: 10.3390/jcm15124588 (PMC13301518; doi:10.3390/jcm15124588)
Supplement: Supplementary file 1 [file jcm-15-04588-s001.zip › Supplementary Table S2.pdf]

STROBE Checklist for Observational Cohort Studies

Manuscript title: "Drone-Induced Midfacial Blast Injuries: Early Reconstruction and 5-Year Outcomes from a Single-Center Cohort"

Section/Item	Recommendation	Reported on Page No.	Comments
Title and Abstract	Indicate study design in title/abstract	1-2	Retrospective analytical cohort study identified in abstract
Introduction	Explain scientific background and rationale	3-4	Background and rationale provided
Objectives	State specific objectives	4	Aim clearly stated
Study Design	Present key study design elements	4	Retrospective single-center cohort
Setting	Describe setting, locations, dates	4	Yerevan, Armenia; Sept-Nov 2020
Participants	Eligibility criteria and selection	5	Inclusion/exclusion criteria reported
Variables	Clearly define outcomes and predictors	5-18	Clinical outcomes and reconstructive variables described
Data Sources	Describe methods of assessment	6	Clinical and radiological evaluation described
Bias	Describe efforts to address bias	25	Limitations and selection bias discussed
Study Size	Explain study size	5	41 eligible patients included
Quantitative Variables	Explain handling of quantitative variables	11-13	Descriptive statistics reported
Statistical Methods	Describe statistical methods	11	Chi-square/Fisher exact tests used
Participants Results	Report participant numbers	5	Flow clarified with exclusions
Descriptive Data	Describe participant characteristics	6-7	Demographics and injury patterns reported
Outcome Data	Report outcomes	13-18	Functional and reconstructive outcomes reported
Main Results	Provide estimates and precision	13	P-values reported
Discussion	Summarize key results	19-25	Key findings summarized
Limitations	Discuss limitations	Before	Dedicated limitations

		Conclusions	section added
Interpretation	Provide cautious interpretation	19-25	Findings interpreted in clinical context
Generalisability	Discuss external validity	25	Limitations/generalizability discussed
Funding	Describe funding source	26	No external funding
