# Supplementary figures and images for: Drone-Induced Midfacial Blast Injuries: Early Definitive Reconstruction and 5-Year Outcomes from a Single-Center Cohort
Source: J Clin Med. 2026 Jun 12;15(12):4588. doi: 10.3390/jcm15124588 (PMC13301518; doi:10.3390/jcm15124588)

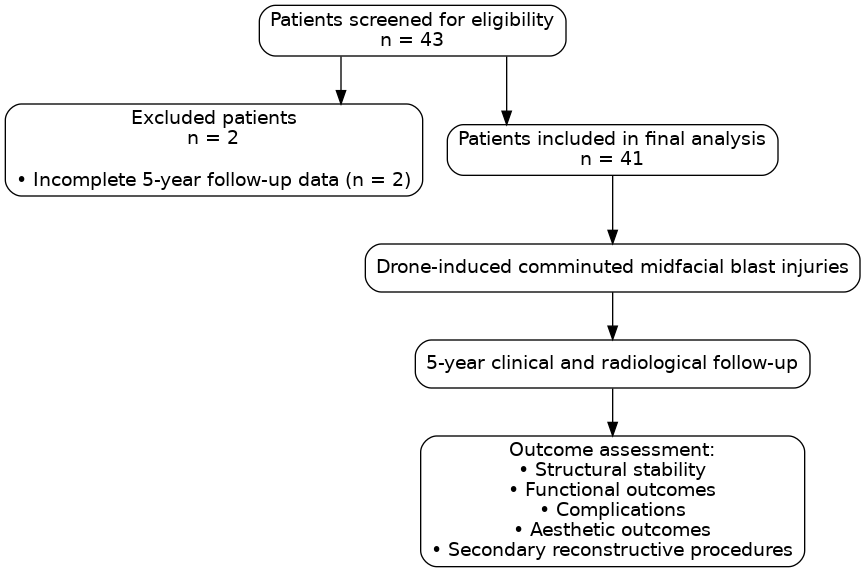

Supplement: Supplementary file 1 [file jcm-15-04588-s001.zip › Supplementary Figure S1.png]
